# Combined use of super-resolution ultrasound imaging and shear-wave elastography for differential diagnosis of breast masses

**DOI:** 10.3389/fonc.2024.1497140

**Published:** 2024-12-20

**Authors:** Yu-Meng Lei, Chen Liu, Hai-Man Hu, Nan Li, Ning Zhang, Qi Wang, Shu-E Zeng, Hua-Rong Ye, Ge Zhang

**Affiliations:** ^1^ Department of Medical Ultrasound, China Resources & Wisco General Hospital, Wuhan University of Science and Technology, Wuhan, China; ^2^ Medical College, Wuhan University of Science and Technology, Wuhan, China; ^3^ Department of Electrical and Electronic Engineering, Hubei University of Technology, Wuhan, China; ^4^ Department of Medical Ultrasound, Hubei Cancer Hospital, Tongji Medical College, Huazhong University of Science and Technology, Wuhan, China; ^5^ Department of Breast Surgery, Hubei Cancer Hospital, Tongji Medical College, Huazhong University of Science and Technology, Hubei Provincial Clinical Research Center for Breast Cancer, Wuhan Clinical Research Center for Breast Cancer, Wuhan, China; ^6^ Department of Cardiovascular Medicine, Wuhan Asia Heart Hospital, Wuhan, China

**Keywords:** breast masses, ultrasound imaging, super-resolution ultrasound, shear-wave elastography, differential diagnosis

## Abstract

**Objectives:**

Shear-wave elastography (SWE) provides valuable stiffness within breast masses, making it a useful supplement to conventional ultrasound imaging. Super-resolution ultrasound (SRUS) imaging enhances microvascular visualization, aiding in the differential diagnosis of breast masses. Current clinical ultrasound diagnosis of breast cancer primarily relies on gray-scale ultrasound. The combined diagnostic potential of tissue stiffness and microvascular characteristics, two critical tumor biomarkers, remains insufficiently explored. This study aims to evaluate the correlation between the elastic modulus, assessed using SWE, and microvascular characteristics captured through SRUS, in order to evaluate the effectiveness of combining these techniques in distinguishing between benign and malignant breast masses.

**Materials and methods:**

In this single-center prospective study, 97 patients underwent SWE to obtain parameters including maximum elasticity (Emax), minimum elasticity (Emin), mean elasticity (Emean), standard deviation of elasticity (Esd), and elasticity ratio. SRUS was used to calculate the microvascular flow rate and microvessel density (MVD) within the breast masses. Spearman correlation analysis was used to explore correlations between Emax and MVD. Receiver operating characteristic curves and nomogram were employed to assess the diagnostic efficacy of combining SRUS with SWE, using pathological results as the gold standard.

**Results:**

Emax, Emean, Esd, and MVD were significantly higher in malignant breast masses compared to benign ones (*p* < 0.001), while Emin was significantly lower in malignant masses (*p* < 0.05). In Spearman correlation analysis, Emax was significantly positively correlated with MVD (*p* < 0.01). The area under the curve for SRUS combined with SWE (0.924) was significantly higher than that for SWE (0.883) or SRUS (0.830) alone (*p* < 0.001), thus indicating improved diagnostic accuracy. The decision curve analysis of the nomogram indicated that SWE combined with SRUS model had a higher net benefit in predicting breast cancer.

**Conclusions:**

The MVD of the breast mass shows a significant positive correlation with Emax. By integrating SRUS with SWE, this study proposes a novel diagnostic approach designed to improve specificity and accuracy in breast cancer detection, surpassing the limitations of current ultrasound-based methods. This approach shows promise for early breast cancer detection, with the potential to reduce the need for unnecessary biopsies and improve patient outcomes.

## 1 Introduction

Breast cancer is a significant global health concern, recently surpassing lung cancer as the most commonly diagnosed cancer worldwide (1). It poses a severe threat to the health of women and remains a critical public health challenge. The current gold standard for diagnosing breast cancer involves invasive procedures, which carry the risk of complications and can damage healthy tissues (2).

Conventional ultrasound imaging is a widely used method for diagnosing breast masses (3, 4), However, its ability to distinguish between benign and malignant masses is limited (5). B-mode ultrasound can provide information about the size, shape, boundary, and internal characteristics of breast masses but is insufficient for detecting blood flow within these masses (6). To supplement B-mode ultrasound, color Doppler flow imaging (CDFI) is used to visualize blood flow distribution within breast masses. However, the overlapping imaging features of benign and malignant masses often necessitate further follow-up and biopsies. CDFI can only show the diameter of >0.2mm vessels and relatively high blood flow rates (>1cm/s) (7, 8). Due to the physical diffraction limit, the spatial resolution of conventional ultrasound imaging, including contrast-enhanced ultrasound (CEUS), which is widely used in clinical practice, is limited by the ultrasound wavelength, and cannot distinguish targets smaller than half a wavelength (8–11). Although it is possible to reduce the wavelength and thus improve the spatial resolution by increasing the ultrasound frequency, the increase in attenuation inevitably induces a decrease in penetration depth, which makes balancing resolution and depth challenging.

The emergence of super-resolution ultrasound (SRUS) imaging technology has revolutionized breast cancer diagnostics. SRUS breaks the acoustic diffraction limit to improve spatial resolution, visualize microvascular, and detect low-speed blood flow (12, 13). It can achieve up to ten-fold improvement in spatial resolution compared to conventional ultrasound imaging in theory (14). By locating individual microbubbles at the sub-wavelength level and tracking their displacement (11, 15), SRUS creates super-resolution velocity maps (SRVM) to observe more microvascular details in breast masses. This technique has been successfully performed on humans (16–19). Moreover, based on microvessel density (MVD) and microvascular flow rate (MFR), it offers valuable information for medical diagnosis of breast cancer (20–22).

Tissue elastic modulus, determined by tumor-associated matrix and collagen, plays a crucial role in the diagnosis and prognosis of tumor aggressiveness (23). Shear-wave elastography (SWE) is a technique that quantitatively measures tissue elastic modulus value and provides a potentially valuable tool to help differentiate benign from malignant breast masses (24).While SWE has demonstrated effectiveness in differentiating masses based on stiffness, it does not capture the microvascular characteristics critical for understanding tumor behavior (25, 26).

By combining SRUS’s microvascular visualization with SWE’s quantitative stiffness measurements, our approach addresses the limitations of single ultrasound modalities that provide incomplete diagnostic information. Previous studies have explored the combination of B-mode ultrasound with SWE (5), SWE with CEUS (27), and B-mode ultrasound with CEUS (28) in breast cancer diagnosis. However, the combination of the tissue elasticity assessment of SWE and the microvascular imaging capabilities of SRUS are not well studied for breast cancer diagnosis, which distinguishes our study from prior work. Our study aims to develop a novel approach for the early diagnosis of breast cancer by integrating the microvascular and stiffness characteristics of breast masses. We also aim to investigate the correlation between Emax and MVD, and to assess the differential diagnosis efficacy of the combination of SRUS and SWE in benign and malignant breast masses, providing a more comprehensive diagnostic method and represents a meaningful contribution to the field.

## 2 Methods

### 2.1 Patient population

**Figure 1:**
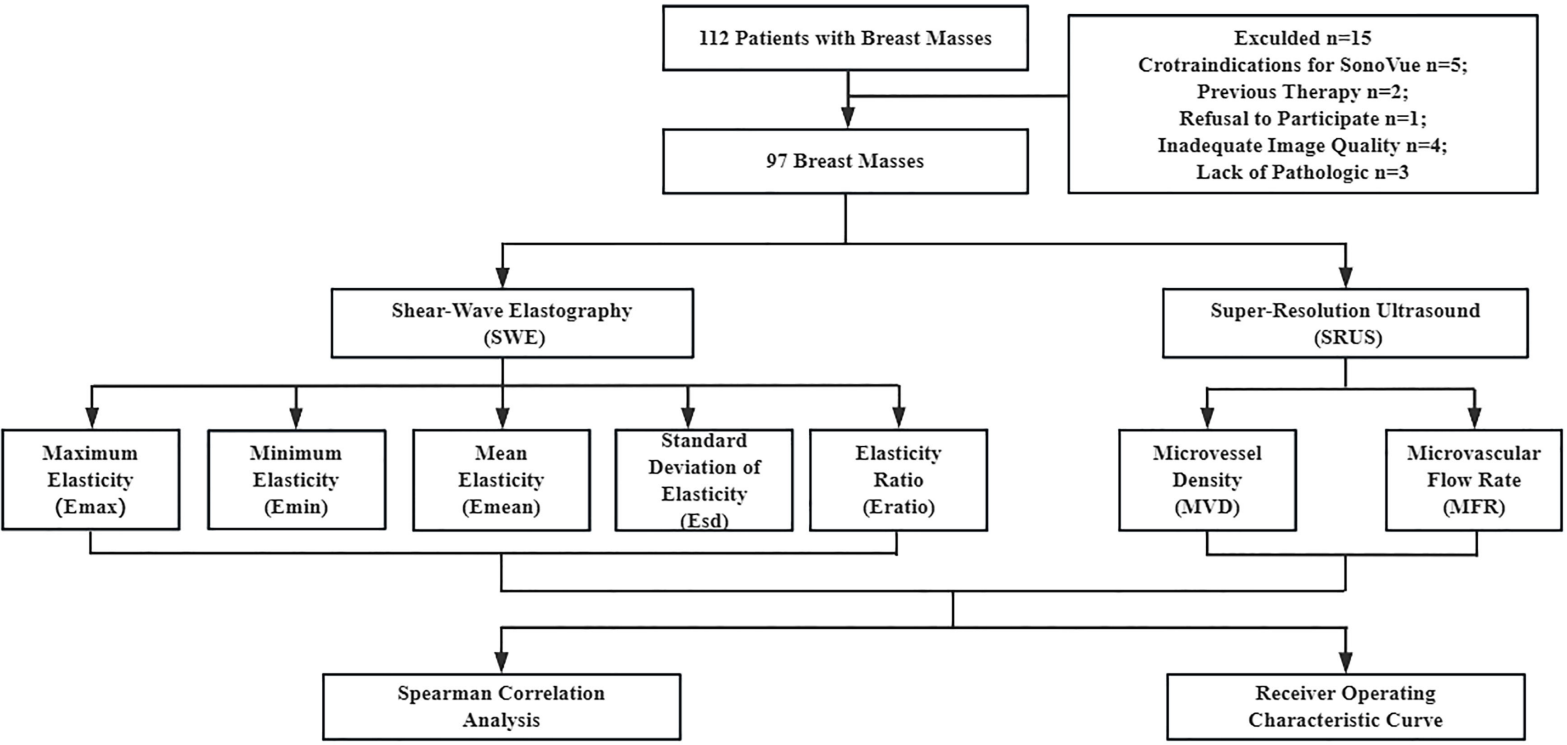
Patient enrollment and data processing pipeline.

This prospective study was approved by the Institutional Review Board of China Resources & Wisco General Hospital. All the patients signed an informed consent form prior to the examination. 97 female patients (aged > 18 years) with breast masses were included in this study between October 2022 and May 2024. In cases where patients had more than one suspicious mass, the most suspicious-appearing mass was selected. Patients were excluded if they had contraindications to ultrasound contrast agents, a history of previous treatment, mental disorders, or any severe cardiovascular or cerebrovascular conditions. Additionally, those with hepatic or renal disease, or who were pregnant, were also excluded from the study. B-mode ultrasound, CEUS, and SWE examinations were performed for all patients. A SonoVue microbubble contrast agent (Bracco, Milan, Italy) was used. Furthermore, all patients underwent surgical excisions to obtain histopathologic results. **Figure 1** illustrates the processes of patient registration and data processing.

### 2.2 Clinical data acquisition

The ultrasound examinations were conducted by a specific radiologist with over 15 years of experience. For real-time ultrasound image monitoring and data collection, a commercial ultrasound system (Resona R9T; Mindray Biomedical Electronics Co., Ltd., Shenzhen, China) and an L11-3U linear array probe with 3.0–10.0 MHz bandwidth were employed. Patients were provided with instructions to assume a supine position, raise their arms, and perform abduction movements during the scanning procedure to ensure comprehensive exposure of the breast and axilla. Furthermore, patients were instructed to maintain a state of quiet respiration during the examination.

The probe was adjusted to show the maximum diameter of the breast mass and ensure that the surrounding tissues were clearly visible. After examining the sonographic characteristics of the breast mass with B-mode ultrasound, the probe was held perpendicular to the examination location, stabilized, and then switched to SWE mode. The whole mass and surrounding breast tissue were selected as two regions of interest (ROI) respectively, and the default values covered the range of 0–180 kPa. The Elasto function key was pressed on the control panel. The quality control badge in the upper right corner of the screen, which showed four stars or more, indicated satisfactory image quality. The image was frozen, and the mass contour was sketched manually along the sliding trackball by the radiologist. Each elastic modulus within the region was automatically calculated by the system, which stored the results as the maximum elasticity (Emax), minimum elasticity (Emin), mean elasticity (Emean), standard deviation of elasticity (Esd), and elasticity ratio (Eratio).

The SRUS image dataset was obtained from CEUS dynamic images after offline processing (see section 2.3 for details). An intravenous injection of a 0.5mL microbubble contrast agent was administered using a 19-gauge needle. The average frame rate was about 80 Hz. A mechanical index (MI) of 0.08 was used to avoid microbubble destruction during the CEUS examinations. The microbubble signal within the breast mass was observed and tracked using B-mode and CEUS dual-mode imaging. During imaging, patients were asked to hold their breath. Then CEUS images were gathered, and additional SRUS processing was performed on the CEUS images to facilitate the calculation of MVD and MFR as follows.

### 2.3 Ultrasound image processing

SRUS image offline analysis was carried out using MATLAB software (MathWorks Inc., Natick, MA, USA). SRUS technology used ultrafast ultrasonic plane wave coherent composite imaging mode to collect the acoustic state of ultrasound microbubbles in microvessels continuously, established two-stage motion correction to correct tissue motion. Then, the post-processing of the SRUS algorithm was carried out.

SRUS used the acoustic response of the microbubble contrast agent to visualize the microvascular system. In this study, locating isolated microbubble signals first required singular value decomposition (SVD) to separate tissue and blood flow signals. The SVD method was first used to transmit and collect many pulse-echo signals and then carried out SVD to remove the tissue signals with large singular values to realize the extraction of microbubble signals. The spatial coordinates of the centroids of the microbubbles were extracted one by one by deconvolution of each source from the predicted Gaussian point spread function. The centroid location was to find the center of the microbubbles by calculating the intensity-weighted centroids of each extracted microbubble signal. With the superposition of multiple frames, SRUS rendering was realized.

MVD was defined as tracked microbubble area divided by the ROI area of mass. ROI was manually constructed on MATLAB based on breast mass contours on B-mode and SRUS images. To calculate the MFR, the tracking method calculated the best correlation bubble signal in the search window between adjacent images. Each microbubble detected in frame F and each microbubble in frame F+1 should be recorded in the search window. The study set the frame rate to 80 Hz and 700 microns as the maximum search window. For each signal in frame F, if the maximum normalized cross-correlation value in frame F+1 was higher than a determined threshold of 0.9, the pairing signal in frame F+1 was identified.

### 2.4 Statistical analysis

The Statistical Package for the Social Sciences version 26.0 (SPSS Inc., Chicago, IL, USA), the R Software (version 4.2.1; R Foundation for Statistical Computing, Vienna, Austria), and GraphPad Prism version 10.1.2 (GraphPad, Inc., San Diego, CA) were used to perform statistical analyses. T-tests, chi-square tests, and Mann-Whitney U tests were employed to compare statistical differences between the benign and malignant groups. Independent variables were screened, confounding factors were eliminated, and the remaining parameters were analyzed using multivariate binary logistic regression. Correlation analysis was conducted within this regression model, selecting variables significant in univariate analysis as independent variables to construct the binary logistic regression equation and determine the cutoff values. Receiver operating characteristic (ROC) curves and area under the curve (AUC) analyses were used to assess the diagnostic efficacy of SWE, SRUS, and their combined application for differentiating benign from malignant breast masses. Sensitivity, specificity, accuracy, positive predictive value (PPV), and negative predictive value (NPV) of the quantitative parameters were obtained via ROC analysis. The dataset was randomly divided into training and validation cohorts at a 7:3 ratio. The training cohort was used for binary logistic regression analysis to identify variables included in the nomogram, while the validation cohort was used for result validation. Nomograms and calibration plots were generated using the rms package. The rms package in R provides tools for building and validating regression models with an emphasis on survival analysis, logistic regression, and other predictive modeling techniques, as well as generating calibration plots and nomograms. The Hosmer-Lemeshow test was used to assess the model’s goodness-of-fit, and decision curve analysis (DCA), conducted with the rmda package, evaluated net benefits within the threshold probability range. The rmda package supports DCA, a method to evaluate the clinical utility of prediction models by calculating net benefits across a range of threshold probabilities. A scatter plot assessed the correlation between Emax and MVD. Statistical significance was defined as p < 0.05, with significance levels noted at 0.05 (*) and 0.01 (**) (two-tailed).

## 3 Results

### 3.1 Ultrasound images of breast masses

**Figure 2:**
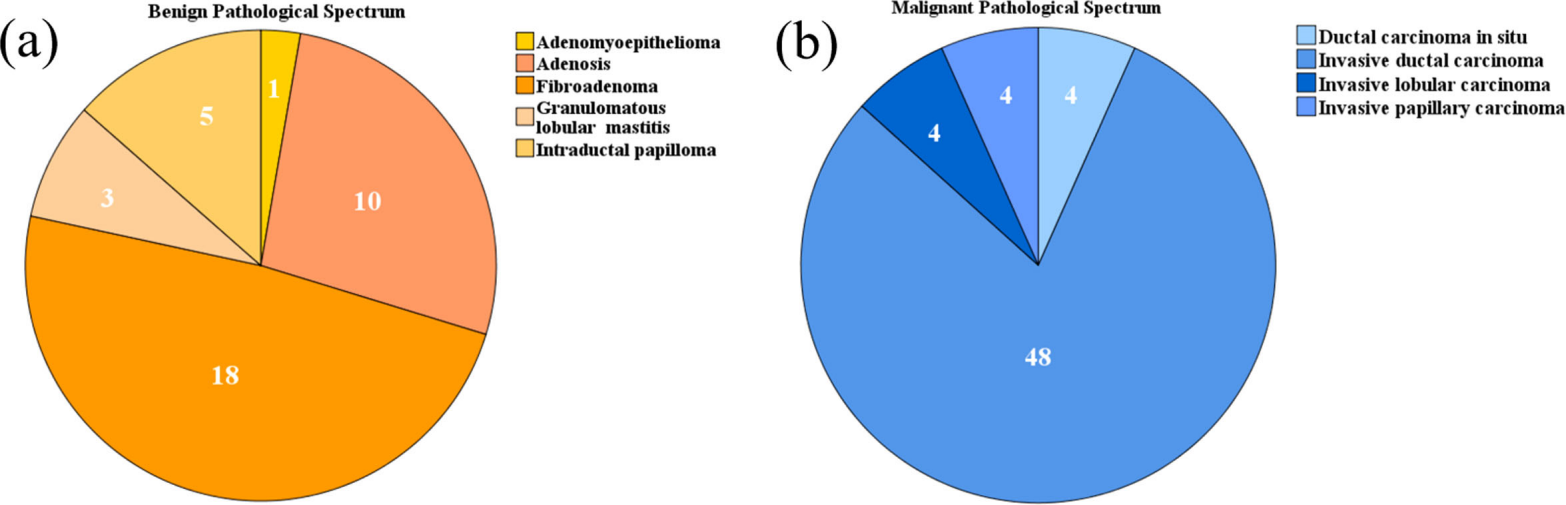
Pie charts depicting the pathological spectra of breast masses: **(A)** Benign pathological spectrum, **(B)** Malignant pathological spectrum.

**Figure 3:**
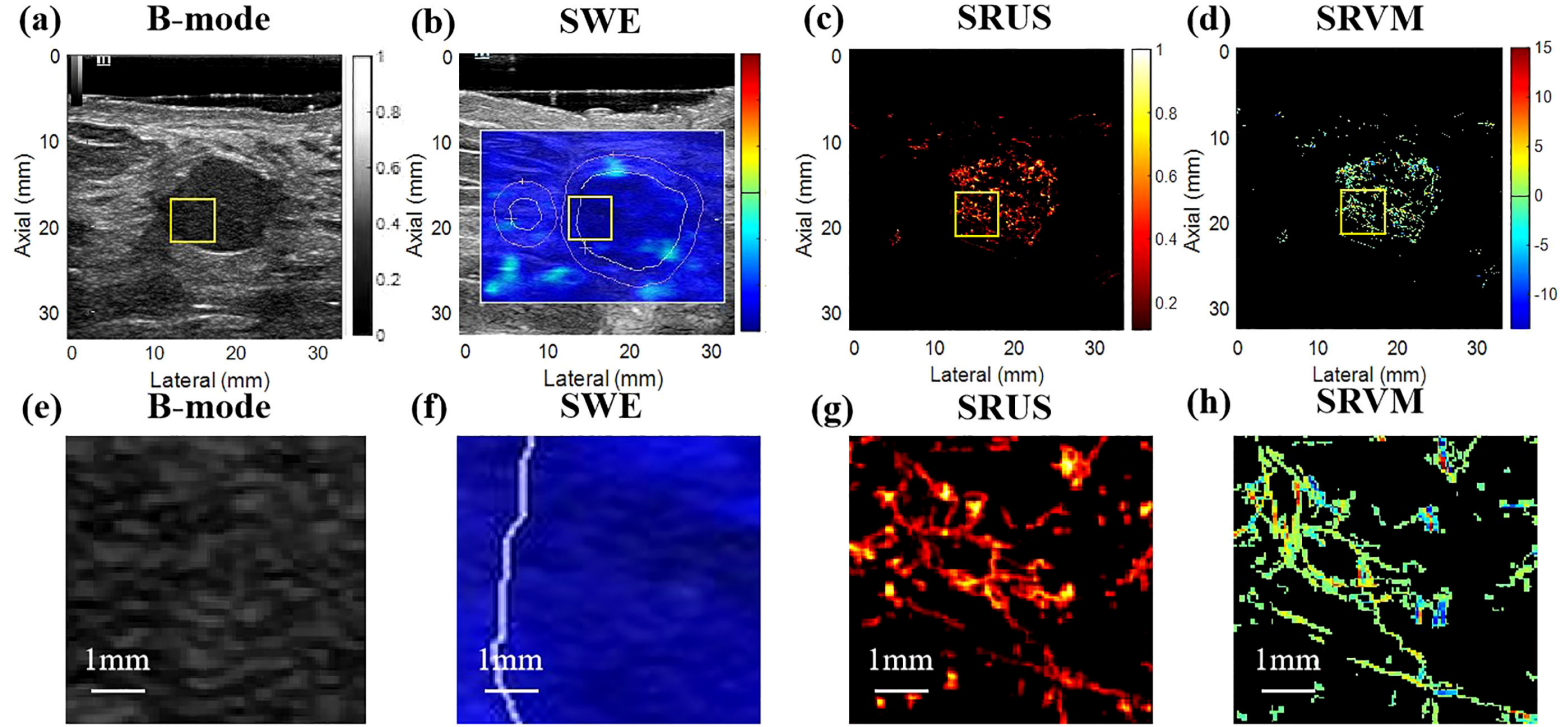
Ultrasound images of benign breast masses. **(A)** B-mode; **(B)** shear-wave elastography; **(C)** super-resolution ultrasound imaging; **(D)** super-resolution velocity map. **(E–H)** were the details of the breast mass between **(A)** B-mode, **(B)** shear-wave elastography, **(C)** super-resolution ultrasound imaging, and **(D)** super-resolution velocity map, as shown in the yellow box in the figure.

**Figure 4:**
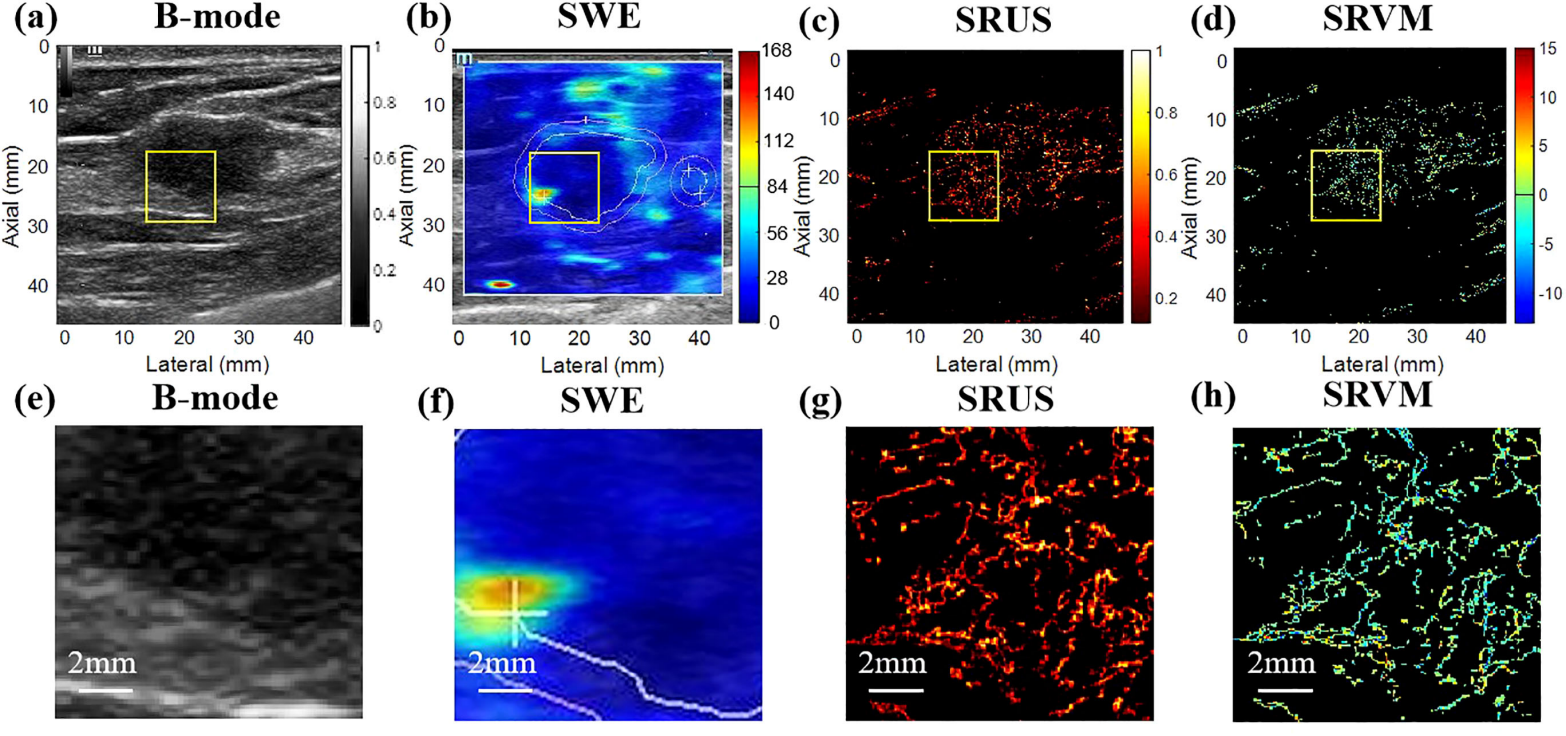
Ultrasound images of malignant breast masses. **(A)** B-mode; **(B)** shear-wave elastography; **(C)** super-resolution ultrasound imaging; **(D)** super-resolution velocity map. **(E–H)** were the details of the breast mass between **(A)** B-mode, **(B)** shear-wave elastography, **(C)** super-resolution ultrasound imaging, and **(D)** super-resolution velocity map, as shown in the yellow box in the figure.

The benign and malignant pathological spectra of the breast cases are shown in **Figure 2**. **Figures 3**, **4** showed B-mode, SWE, SRUS, and SRVM images of a patient with a benign and a malignant breast mass, respectively. The image below (e-h) corresponds to the local magnification in the box of the ultrasound image described above (a-d). SRUS demonstrated improved temporal and spatial resolutions while maintaining adequate penetration depth and visual field, enabling observation of microvascular at the micron scale. In the SRVM, red and blue indicated opposite directions and relatively high flow velocity, respectively, while yellow showed relatively low flow velocity, providing additional microvascular velocity information.

### 3.2 Comparison of clinical information and quantitative parameters

According to the data presented in **Table 1**, the findings of this study demonstrated the presence of statistically significant variations in age, size, position, Emax, Emin, Emean, Esd, and MVD between benign and malignant masses (*p* < 0.05), with malignant masses having greater values of Emax, Emean, Esd, and MVD, while benign masses had greater values of Emin. To further evaluate the diagnostic potential of these quantitative parameters, the study performed binary logistic regression analyses, and the results were presented in **Table 2**. Only Emax and MVD showed statistical differences. The higher OR value of MVD compared with Emax indicated that MVD was more relevant in the differential diagnosis of breast masses.

**Table 1:** Summary of clinical information from examined patients and the corresponding ultrasound characterizations of breast masse.

Parameters	Benign	Malignant	t//χ²/z	*p*
Number of masses	37	60		
Age	45.08 ± 10.69	52.62 ± 10.81	-3.348	0.001
Size (cm)	1.62 (1.21, 2.74)	2.71 (1.96, 4.01)	-3.869	<0.001
Position			-2.027	0.038
Right	21 (56.7)	26 (43.3)		
Left	16 (43.2)	34 (56.7)		
Emax (kPa)	81.66 (52.26, 103.24)	189.58 (97.59, 267.88)	-5.667	<0.001
Emin (kPa)	4.05 (2.06, 7.61)	1.76 (0.10, 5.40)	-2.700	<0.05
Emean (kPa)	21.75 (14.69, 31.29)	32.22 (23.91, 51.09)	-3.721	<0.001
Eratio	1.42 (0.98, 2.14)	1.69 (1.23, 2.63)	-1.597	>0.05
Esd (kPa)	10.10 (6.78, 16.36)	21.75 (13.04, 32.32)	-4.969	<0.001
MVD (%)	1.01 (0.27, 3.44)	5.15 (2.52, 8.23)	-5.433	<0.001
MFR (mm/s)	10.25 (8.46, 12.92)	11.38 (10.24, 12.75)	-1.764	>0.05

**Table 2:** Binary logistic regression analysis results of quantitative parameters in diagnosing benign and malignant breast masses.

Parameters	β	S. E	Wald	*p*	OR	95% C.I. for OR	
						Lower	Upper
Emax	0.035	0.013	6.951	0.008	1.036	1.009	1.063
MVD	0.442	0.140	9.919	0.002	1.555	1.182	2.047
Constant	-3.608	0.991	13.258	0.001	0.027		

### 3.3 Relationship between Emax and MVD

**Figure 5:**
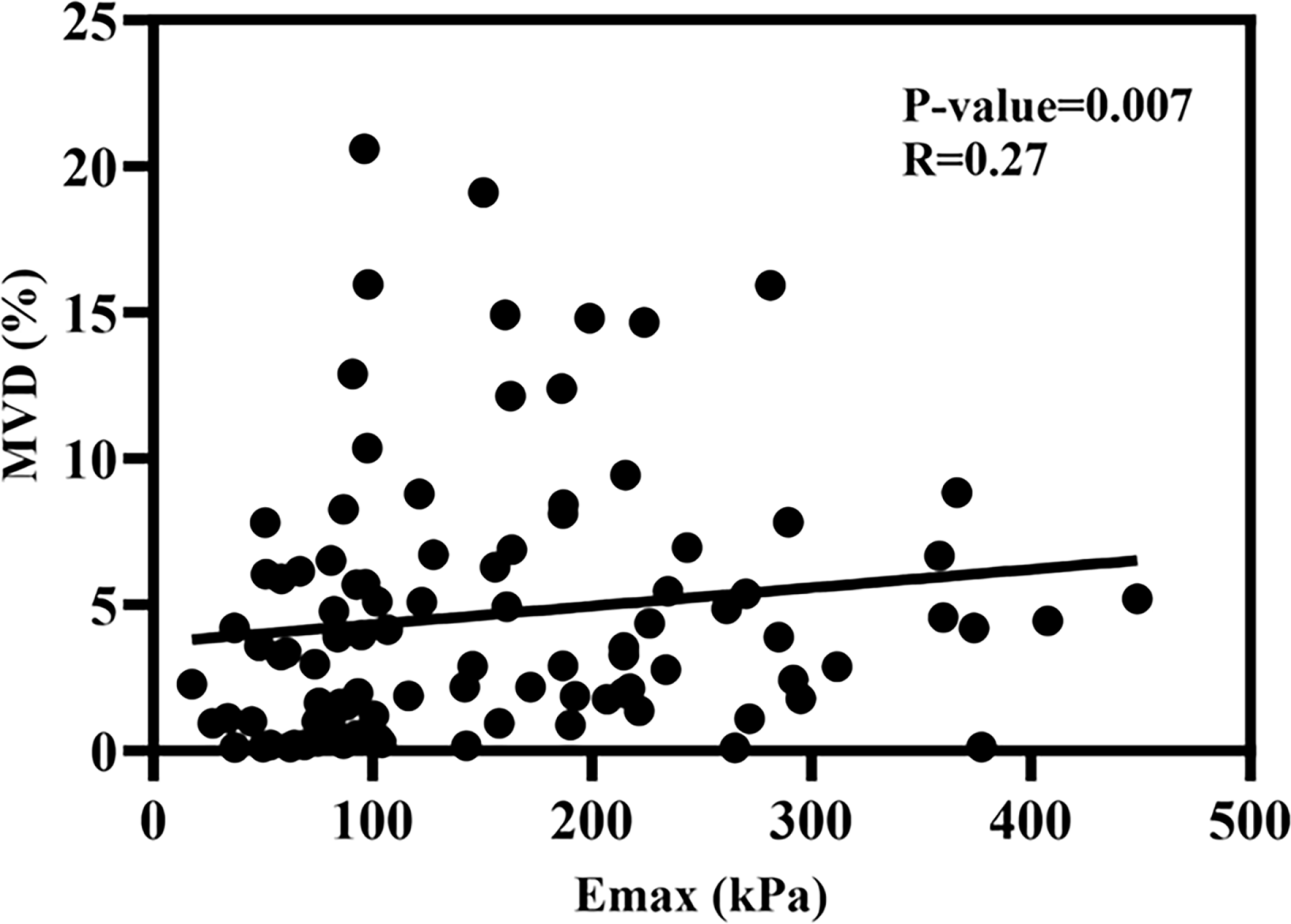
The scatter plot of the correlation coefficient between Emax and MVD.

This research employed a scatter plot to assess the association between Emax and MVD. The scatter plot and fitting curve of correlation between Emax and MVD are shown in **Figure 5**, and the result showed that Emax and MVD were significantly positively correlated, with a correlation coefficient of approximately 0.27 (*p* < 0.01).

### 3.4 Diagnostic performance of SWE and SRUS combination

**Figure 6:**
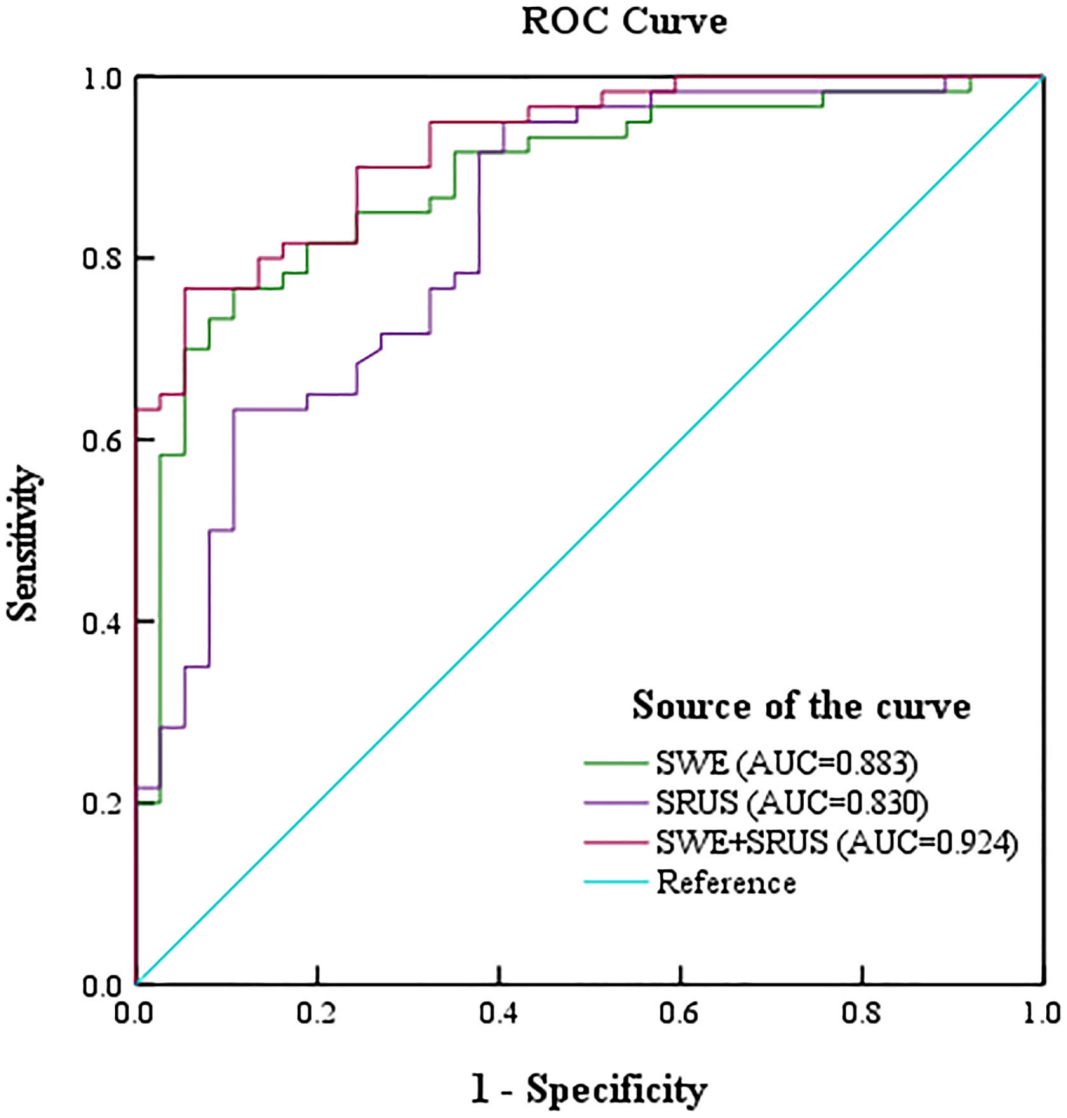
The receiver operating characteristic curve was drawn to show the diagnostic performance of shear-wave elastography, super-resolution ultrasound imaging, and their combination in distinguishing benign and malignant breast masses.

ROC analysis evaluated the diagnostic efficacy of SWE, SRUS, and the combination of SWE and SRUS in differentiating benign from malignant breast masses, including parameters such as sensitivity, specificity, accuracy, PPV, NPV, cutoff values, and AUC. Binary logistic regression analysis yielded regression equations for the SWE and SRUS groups as follows: for the SWE group, logit(p) = -1.857 + 0.036Emax - 0.225Emin; for the SRUS group, logit(p) = -1.112 + 0.454MVD, with cutoff values of 0.639 and 0.375, respectively. The regression equation and cutoff value for the combined SWE and SRUS group were logit(p) = -3.608 + 0.442MVD + 0.035Emax and 0.714, respectively. As presented in **Table 3**, the SWE group demonstrated an AUC of 0.883 (95% CI: 0.815-0.952; *p* < 0.001), while the SRUS group had an AUC of 0.830 (95% CI: 0.745-0.914; *p* < 0.001). Notably, the combined SWE and SRUS approach yielded the highest AUC at 0.924 (95% CI: 0.875-0.973; *p* < 0.001), outperforming the use of SWE or SRUS alone. Consequently, the combined SWE and SRUS approach achieved an accuracy of 83.5%, sensitivity of 76.7%, and specificity of 94.6%. **Figure 6** illustrates the ROC curves for SWE, SRUS, and their combined application, highlighting the superior diagnostic performance of the combined SWE and SRUS group with the largest AUC.

**Table 3:** To evaluate the diagnostic efficacy of SWE, SRUS, and combination in differentiating benign and malignant breast masses.

Group	Sensitivity (%)	Specificity (%)	Accuracy (%)	PPV (%)	NPV (%)	Cutoff Value	AUC	95% C.I.
SWE	76.7	89.2	79.4	81.7	75.7	0.639	0.883	0.815-0.952
SRUS	95.0	59.5	72.2	75.0	67.6	0.375	0.830	0.745-0.914
SWE+SRUS	76.7	94.6	83.5	88.3	75.7	0.714	0.924	0.875-0.973

### 3.5 Nomogram construction of SWE and SRUS combination

**Figure 7:**
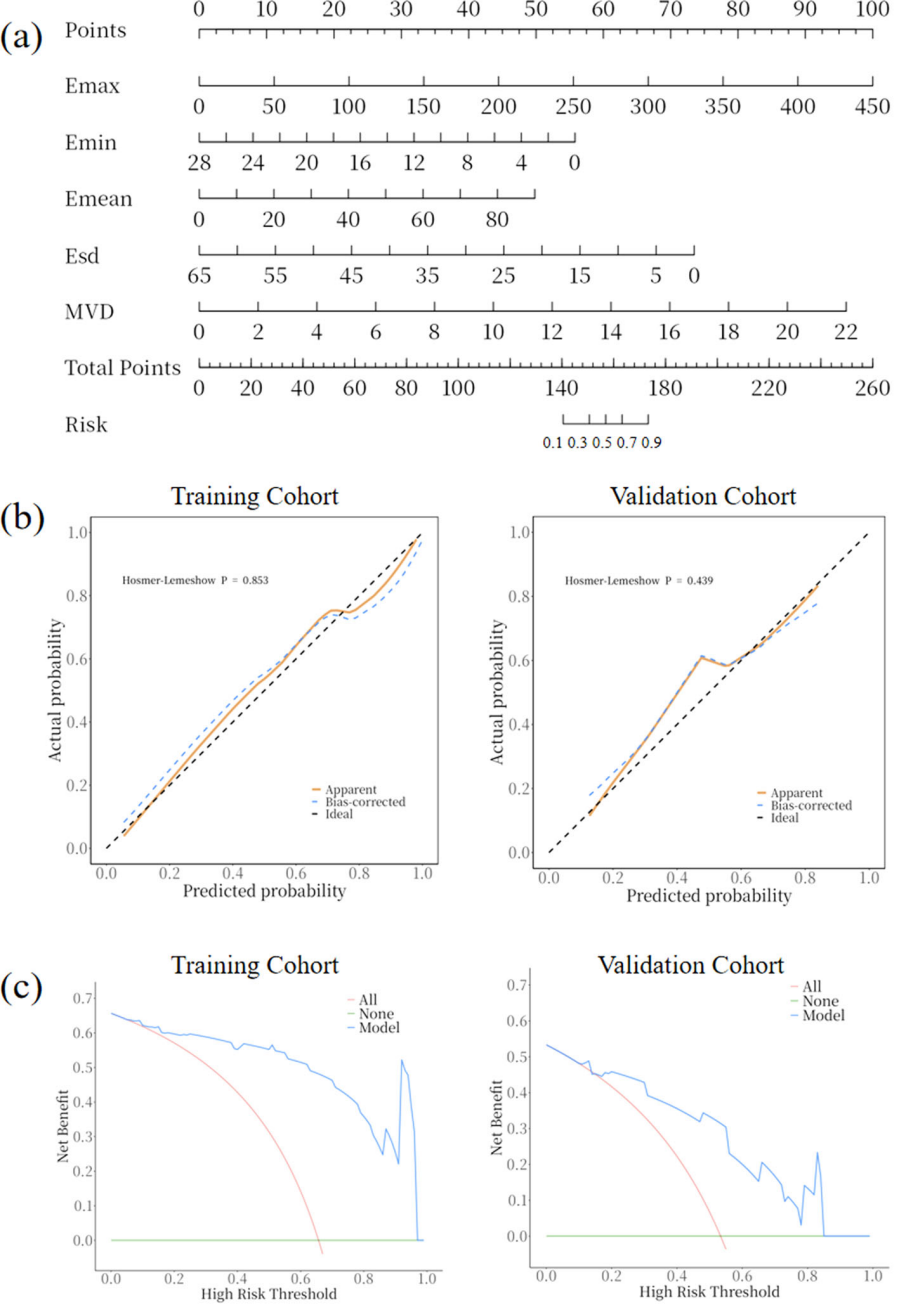
Nomogram, calibration curve, and decision curve analysis for the SWE combined with SRUS model in predicting breast cancer. **(A)** Nomogram; **(B)** Calibration curves for the training and validation cohorts; **(C)** Decision curve analysis for the training and validation cohorts.

A multiparameter prediction model combining SWE and SRUS was developed and visualized as a nomogram to estimate breast cancer risk, as shown in **Figure 7A**. For example, consider a patient with the following parameter values: an Emax of 80.0 kPa (equivalent to 18 points on the nomogram), an Emin of 5.0 kPa (45 points), an Emean of 25.0 kPa (13 points), an Esd of 8.0 kPa (62 points), and an MVD of 1.5% (8 points). The cumulative score for this patient is 146 points. According to the nomogram, a total score of 146 points corresponds to an estimated breast cancer risk of 22%. **Figure 7B** The calibration curve for the nomogram demonstrated good agreement between the model’s predictions and actual observations. In both the training and validation cohorts, the Hosmer-Lemeshow test yielded p-values greater than 0.05 (p = 0.853 and p = 0.439), indicating a good fit of the nomogram to the data. The DCA of the nomogram is shown in **Figure 7C**. The results indicated that the SWE combined with SRUS model provided a higher net benefit in predicting breast cancer than either a treat-all-patients or treat-none approach across nearly all threshold probabilities in both the training and validation cohorts.

## 4 Discussion

The study involved 97 female patients with breast masses who underwent B-mode ultrasound, SWE, and CEUS examinations. The study included five distinct types of benign lesions and four distinct types of malignant lesions, categorized based on pathological subtypes. In our study, we evaluated the significance of the quantity parameters between SWE and SRUS, and explored the correlation between Emax and MVD. The results demonstrated that MVD exhibited the highest differential diagnostic value among the parameters evaluated, with a significant positive correlation to the Emax of the breast mass. Furthermore, we combined microvascular imaging quantification from SRUS with quantitative stiffness measurements from SWE to assess breast cancer and evaluated the diagnostic performance of this integrated approach. To evaluate the diagnostic accuracy, ROC curves, a nomogram, and DCA were utilized, highlighting the importance of these modalities in improving the diagnosis of breast masses and guiding clinical decision-making. The ROC analysis revealed that the diagnostic efficacy of the combined SWE and SRUS approach surpassed that of either modality alone. Additionally, DCA demonstrated that the SWE-SRUS combination model offered a higher net benefit in predicting breast cancer. Our integrated approach, combining SWE and SRUS, provides a more comprehensive diagnostic evaluation compared to conventional ultrasound methods.

Previous studies have explored the combined diagnostic efficacy of various ultrasound modalities, such as B-mode ultrasound with CEUS (28), B-mode ultrasound with SWE (5), and CEUS with SWE (27). The integration of different ultrasound techniques has been shown to improve diagnostic accuracy. However, the potential benefits of combining tissue stiffness assessment through SWE with microvascular imaging from SRUS for breast cancer diagnosis remain unclear. Therefore, this study aims to evaluate the clinical applicability and potential of combining SWE and SRUS in the diagnosis of breast cancer.

SWE has been widely used to access breast masses based on tissue elastic modulus, with previous studies confirming its effectiveness in distinguishing between benign and malignant masses (29–31). In this study, the Emax, Emean, and Esd values of malignant breast masses were higher than those of benign masses, while Emin values were lower, consistent with earlier findings (32–34). This may be due to the presence of more elastic fibers and interstitial components in malignant masses, which are densely arranged. In contrast, benign masses often have a looser arrangement of fibrous stroma and glands, a higher mucopolysaccharide content, and a softer texture. For the microvascular quantification within breast masses, SRUS provides high resolution, and considerable depth, and surpasses the diffraction limit in structural imaging, making it applicable for differentiating benign from malignant breast masses. In this study, the quantitative analysis of SRUS parameters revealed that MVD was significantly higher in malignant breast masses compared to benign ones, while MFR did not differ significantly between the two groups. This may be due to malignant masses producing more growth factors, promoting angiogenesis, which increases MVD values. However, neovascularization in malignant tumors is often structurally and functionally abnormal, leading to vessel collapse and blood flow stagnation, which affects the flow rate (35).

The value of MVD affects the biological behavior of breast cancer to some extent, and the biological behavior often determines the physical changes of the tumor, and the change of elastic modulus is also one of the physical changes of the tumor (36, 37). Therefore, the elastic modulus may have some correlation with MVD, which is one of the primary purposes of this study. Because malignant masses usually have high heterogeneity, they exhibit significant regional differences in elastic modulus. And therefore Emax is a quantitative parameter which could better capture this heterogeneity, outperforming other elastic modulus values (38, 39). This study primarily explores the correlation between Emax and MVD. This study found that Emax has a significant positive correlation with MVD, consistent with the conclusions of Jamin et al. and Jugé et al. (40, 41). This may be because increased elastic modulus further activates signaling pathways related to tumor cell proliferation and invasion, thus, angiogenesis and vascular permeability are increased. MVD as a quantitative criterion of angiogenesis in breast tumors will increase accordingly (42–45).

Our study showed that the AUC of SRUS combined with SWE was as high as 0.924, significantly higher than that of SRUS (0.830) or SWE (0.883). And it is worth noting that the combined group has higher specificity. These findings highlight that the combination of SRUS and SWE significantly improves diagnostic performance. ROC analysis showed that the AUC value of the SRUS group was slightly lower than that of the SWE group, which may be related to the selection of four statistically significant parameters Emax, Emin, Emean, and Esd in the SWE group. Only MVD was selected as a statistically significant parameter in the SRUS group. At the same time, the higher specificity of the combined group suggests that it can reduce false-positive cases and accurately exclude benign masses, which is of great value in reducing unnecessary biopsies. Lee et al. showed that combining quantitative parameters of SWE and superb microvascular imaging with B-mode significantly improved the diagnostic efficacy in differentiating benign from malignant breast masses, with an AUC of the combination of 0.849 (46). Chen et al. constructed a predictive model for breast cancer diagnosis using relevant parameters from SWE and CEUS. ROC curve analysis showed that the AUC reached 0.857, with significantly higher accuracy compared to conventional ultrasound (47). Our studies were consistent with the results of previous studies. This highlights the potential of this combined technique as an assessment of benign and malignant breast masses, thereby informing clinical decision-making and improving patient outcomes.

We also developed a nomogram that combines SWE and SRUS to predict breast cancer risk, demonstrating strong performance on the calibration curve and in DCA. This nomogram shows potential clinical value by enhancing net benefit in clinical decision-making.

Although this study included five types of common benign lesions and four types of common malignant lesions, with larger sample sizes and a broader pathological spectrum, the combined SWE and SRUS approach could offer valuable insights for identifying marginal or rare lesions. The approach incurs only the additional cost of an ultrasound contrast agent compared to conventional ultrasound, while offering significant potential to reduce unnecessary biopsies and associated healthcare expenses. Although a detailed cost analysis was not performed, the improved specificity of this method could help minimize misdiagnoses and follow-up interventions, ultimately reducing both patients’ psychological burden and overall healthcare costs.

However, this study has several limitations. The small sample size, limited range of breast disease types, use of only an internal validation cohort, and single-center design highlight the demand for larger sample sizes, a broader pathological spectrum, external validation, and multicenter prospective studies. In future research, we aim to optimize the study design, conduct multicenter prospective studies, and expand the cohort size to ensure more robust and generalizable findings, ultimately enhancing the clinical applicability of our results. Additionally, the two-dimensional (2D) nature of the ultrasound SRUS and SRVM images does not capture out-of-plane microvascular. Previous studies have reported three-dimensional (3D) ultrasound SRUS by acquiring 2D SRUS stacks using a mechanical scanning system to translate a one-dimensional array transducer (11, 13, 48). Advancements in 3D ultrasound SRUS might significantly enhance diagnostic accuracy by capturing the full volumetric context of breast masses, minimizing the risks of sampling bias inherent to 2D imaging. However, commercially available 3D super-resolution ultrasound equipment for clinical application has not yet been developed. To ensure consistency in image acquisition across different ultrasound modes, the maximum diameter section of each breast mass was selected for imaging. However, this approach may introduce potential sampling bias. For instance, local necrosis within the maximum diameter section could lead to decreased stiffness or reduced MVD. Notably, recent studies have demonstrated good repeatability of SRUS across different sections of breast masses (49), though the repeatability of SWE across various sections requires further investigation.

In conclusion, the MVD of the breast mass exhibits a significant positive correlation with Emax. The combination of SRUS and SWE provides substantial advantages for breast cancer detection. This study presents an enhanced approach for the differential diagnosis of common benign and malignant breast masses, improving specificity and accuracy. Moving forward, we aim to further investigate the impact of SWE and SRUS on breast cancer histological types, biopsy rates, and other relevant factors. We believe that with continued research, the value of SRUS and SWE in the early diagnosis of breast cancer will become increasingly evident.

## Data Availability

The original contributions presented in the study are included in the article/supplementary material. Further inquiries can be directed to the corresponding author.
